# Glamour, expression, and consequences of tattoos in radiation treatment

**DOI:** 10.1371/journal.pone.0220030

**Published:** 2019-08-07

**Authors:** Paulina E. Galavis, Nicholas J. Sanfilippo, Indra J. Das

**Affiliations:** 1 Department of Radiation Oncology, NYU Permuletter Cancer Center, NYU Langone Health, New York, NY, United States of America; 2 Department of Radiation Oncology, Weill Cornell Medicine, New York, NY, United States of America; ENEA Centro Ricerche Casaccia, ITALY

## Abstract

It is estimated that approximately 24% of the US population has at least one tattoo. However, tattoo ink ingredients include heavy metals (high atomic number Z) that are not regulated, which can cause skin reactions. This study investigates the dosimetric effects in surface dose due to high-Z elements in tattoo ink under electron beam irradiation. Four commercially available tattoo ink colors, black, red, yellow, and blue were chosen. The elemental composition of the tattoo ink samples was analyzed using X-ray Fluorescence (XRF). An ultrathin-window parallel plate ion chamber was used to measure the surface dose perturbation (ratio of ionizations with and without tattoo ink) for 6 − 20 MeV electron beams. The elemental concentration in the tattoo ink samples showed high-Z elements, with Z ranging from 11 to 92. The dose perturbation ranged from 1.4% up to 6% for the yellow ink for the 6 MeV electron beam, with similar values across the rest of the electron energies, whereas the black, red, and blue inks presented up to 3% dose perturbation for the same range of energies. Based on this initial study, we conclude that commercially available tattoo inks contain large amounts of high-Z metals that may contribute to dose perturbation. Therefore treatment of superficial lesions with electron beams in a tattooed area should be monitored for signs of early skin reaction during radiation therapy treatments.

## Introduction

Tattooing has become a social phenomenon, as it is estimated that approximately 24% of the US population has at least one tattoo [1]. Tattoo ink comes in many colors, from traditional black to almost every color in the light spectrum. These bold colors have genesis in the heavy metals in the ink, which produce the pigmentation [2]. Spectrophotometric analysis of trace metals in tattoo ink has revealed the presence of multiple elements at levels exceeding the traditionally accepted safe limit of 1 *μ*g/g [3]. Interestingly, the US Food and Drug administration (FDA) neither approves nor regulates tattoo ink as they do with medications or medical devices, because the FDA considers tattoo inks to be cosmetic and the pigments used in the inks are considered additives [4]. Therefore, manufacturers are not required to reveal tattoo ink ingredients.

Traditionally, skin cancers and superficial tumors (most common and prevalent in the USA) have been treated with kilovoltage beams to deliver 100% dose to the skin. However, as the availability of kilovoltage beams has decreased in the USA, electron beams have taken their place to treat these patients. It has been shown that there are two intertwined well-studied phenomena [5–12], dose perturbation and electron backscattering. These occur when an electron beam encounters an interface (e.g. a non-water type material) on its path and secondary electrons released in both media change the electron fluence on both sides of the interface, creating a dose perturbation and electron backscatter, which depend on the electron beam energy, particle type, and the atomic number (Z) of the two media.

This study was designed to investigate an unexpected skin toxicity (serious erythema) in a patient who was treated with an electron beam in a partially tattooed area at the level of the left upper chest (between the shoulder and the clavicle). Since the examination of the patient’s chart and dosimetry did not reveal any reason for the skin reaction (much earlier than anticipated), our hypothesis is that the high-Z material in the tattoo ink may have interfered and created dose perturbation when irradiated with the electron beam. To the best of our knowledge, there are no reports studying the possible effects of ionizing radiation on treated tattooed regions.

## Materials and methods

### The tattoo ink composition

Four commercially available tattoo ink colors (Iron Sakura Tattoo Ink, Taiwan) were chosen for this study. Black, red, yellow and blue were selected based on their popularity among tattoo customers and also because these have been reported to produce adverse skin reactions [13, 14]. The ink samples were sent for X-ray Fluorescence (XRF) analysis to determine the elemental composition of the ink. For measurements, 10 mg of each tattoo ink color were smeared on to a 37 mm teflon-filter. Any values of elemental composition found below two times their uncertainties were considered below the lowest detectable level of the instrument for that particular element. The results were sorted in descending order according to the amount of each element expressed in ng/cm^2^. To estimate the amount of each element in the ink samples, we calculated the percentage of each element based on ng/cm^2^ times the 10 mg.

### Dosimetric perturbation

In this study we use the definition of surface dose perturbation as the ratio of ionizations of the ion chamber readings with and without the tattoo ink. This parameter was measured at the surface of an ultrathin-window parallel plate ion chamber, PTW-Markus (sensitive volume of 0.02 cm^3^), positioned at the center of a customized 30×30 cm^2^ solid water slab, placed on top of 5 cm solid water to avoid back-scatter from the linac couch. This setup was chosen to mimic the surface of the skin as the ion chamber has 1 mm thick water-proof material, thus limiting the measure to depths comparable to those of the skin. Given that the dose perturbation is defined as a relative dose, no stopping power correction was deemed necessary to convert readings to dose.

Measurements were taken on a Varian TrueBeam linear accelerator for all the available electron energies in the range of 6-20 MeV, using 100 cm source to skin distance (SSD) and a 10×10 cm^2^ electron cone size. Measurements with the tattoo ink were performed by measuring each ink-drop (measured with a 3 cc syringe) and smearing it over a 1.4 cm diameter on the surface of the ion chamber (properly covered with thin ink resistant paper). Assuming a cylindrical volume, the ink thickness was estimated using *thickness* = *Ink*_*volume*_/(*π* × *r*^2^). The effect of bolus was not evaluated due to the difficulty of measuring with tattoo liquid, which could smear further causing uncertainty on the thickness of the tattoo ink.

### Skin reaction

The patient was CT simulated and planned in Eclipse (Varian Medical Systems) treatment planning system using a 9 MeV electron beam (200 cGy per fraction for 30 fractions) and a 0.5 cm bolus to treat a dermatofibrosarcoma protuberans of the trunk after surgery. Electron Monte Carlo (Version 13.7.14) algorithm was used to calculate the dose. The patient showed a brisk skin reaction early in treatment (600 cGy). Optically Stimulated Luminescence Dosimeters (OSLDs) were used for *in vivo* measurements in order to evaluate the accuracy of the treatment as part of our institutional quality assurance. The OSLD detector was placed at the central axis of the treatment field under the 0.5 cm bolus, a 0.6% difference between the planned and measured dose was found.

## Results

**Fig 1:**
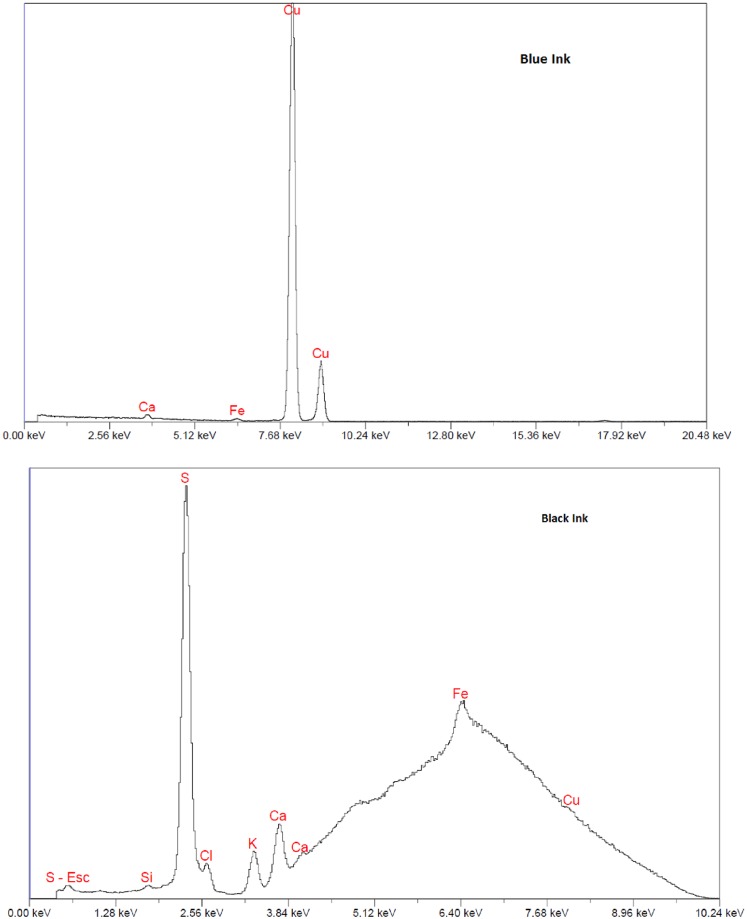
Spectrum of the red and yellow tattoo ink used in this study.

**Fig 2:**
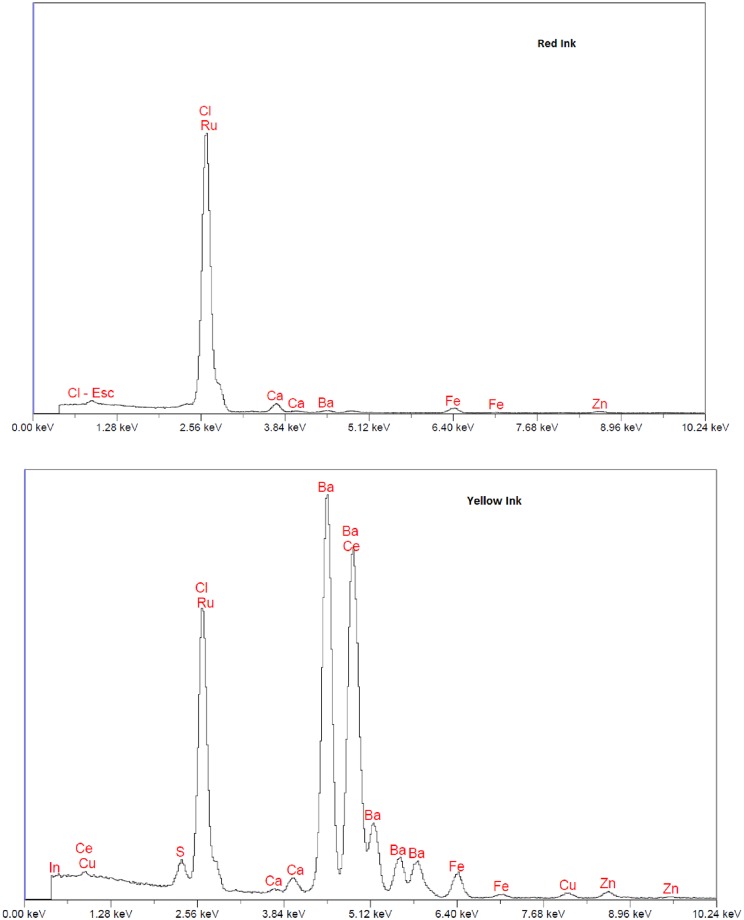
Spectrum of the blue and black tattoo ink used in this study.

**Fig 3:**
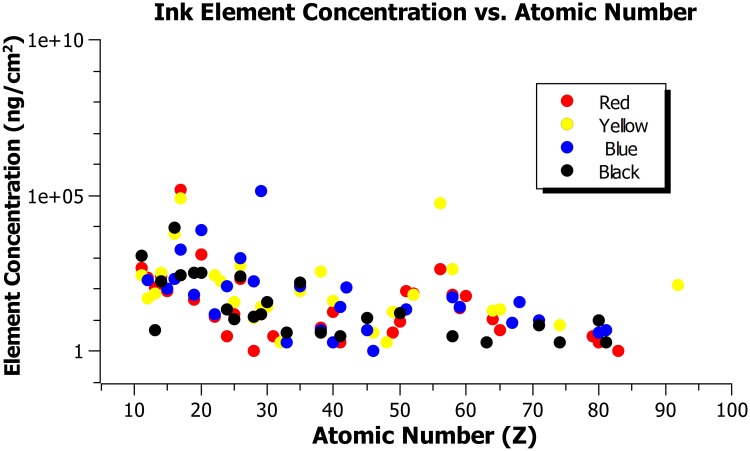
Element concentration for the black, red, blue, and yellow tattoo inks as a function of the atomic number, Z.

**Fig 4:**
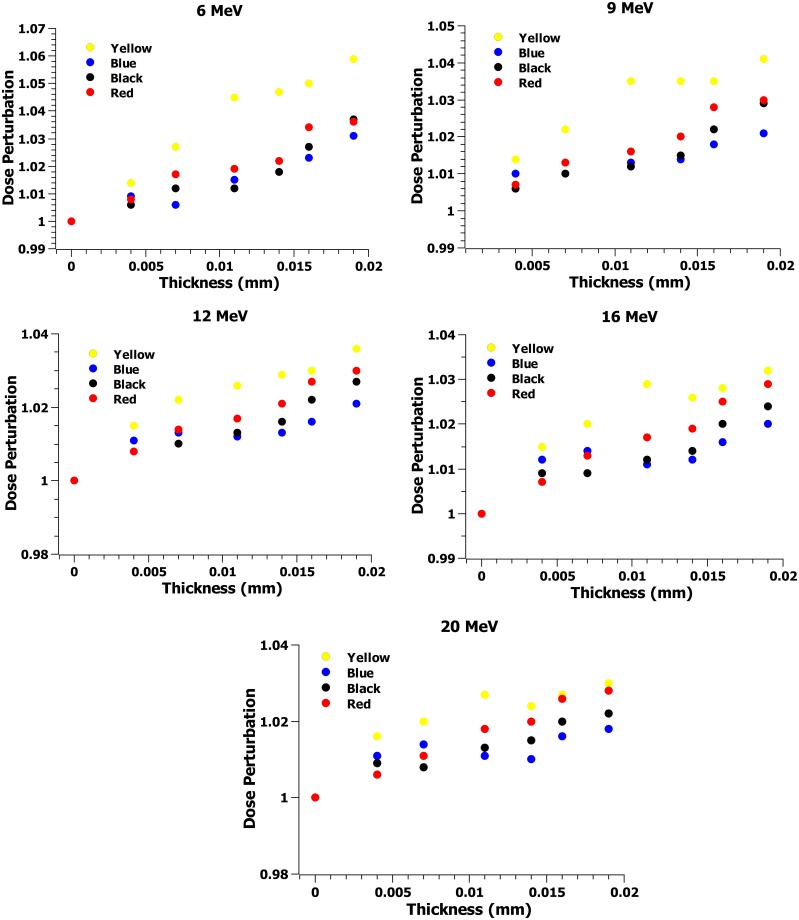
Dose perturbation scatter plots for the black, red, blue, and yellow tattoo ink for 6-20 MeV electron beam energies.

The spectral plots of the tattoo ink studied are shown in Figs 1 and 2. The elemental concentration in the tattoo inks studied as a function of the atomic number is shown in Fig 3, indicating presence of high-Z elements ranging from 11 to 92. Table 1 summarizes the major element concentrations in percentages, the red ink showed the highest atomic number material composition. The dose perturbation (ratio of dose with and without tattoo ink) with electron beams scatter plots for black, red, blue, and yellow tattoo ink for all the electron energies are shown in Fig 4. The dose perturbation ranged from 1.4% up to 6% for the yellow ink for the 6 MeV electron beam, with similar values observed across the rest of the electron energies, whereas the black, red, and blue inks presented up to 3% dose perturbation in the same energy range. We calculated the type A uncertainty on the dose perturbation by looking at the standard deviation of the average readings from the ion chamber measurements. In addition, straightforward error propagation on the dose perturbation gives us a relative standard uncertainty error of < 0.1%. For completenes, an error propagation on the thickness equation gives a relative error about 0.4%. In addition, type B uncertainties were estimated based on manufacturer specifications, and published references [15, 16] as shown in Table 2.

**Table 1:** Major element composition for the tattoo inks.

Ink Color	Major Element	Atomic Number (Z)	Composition (%)
Black	Sulfur	16	77.1
Red	Chlorine	17	97.8
Yellow	Chlorine and Barium	17 and 56	55.6 and 38.2
Blue	Copper	29	92.4

**Table 2:** Type B uncertainties.

Uncertainty Components	Type B(%)
Ion chamber SSD setup [15]	0.10
Ion chamber recombination [16]	0.21
Ion chamber thermal effect [16]	0.11
Field size reproducibility [15]	0.10
Linac stability [15]	0.05
Measurement repeatability (electrometer)	0.05
Combined uncertainty (k = 1)	0.08

## Discussion

Due to the lack of studies on the possible effects of tattooing in radiation therapy, this study was conducted to investigate the dose surface perturbation in electron beams due to the high-Z material content in the tattoo ink. It was found that high-Z components, as shown in Fig 3, are part of the composition of the inks that were analyzed. The impact of these inks in electron beam dosimetry is shown in Fig 4, and it can be seen that the dose pertubation increases with increasing tattoo ink thickness. From this we can infer that there is some dose enhancement resulting from the transit of electrons through thin layers of ink, however no clinical correlation of skin erythema to the small increase in surface dose was measured in this work. The skin reaction could be related to electron backscatter, as it has been shown to play a significant role depending on the material and the electron energy. In addition, there are physiological changes in the skin of a tattooed area that cannot be reproduced in a experiment without biopsy.

This study was limited by the uncertainties on the ink thickness in an actual tattoo applied to the patient’s skin, and the actual concentration of the metals in the skin. A retrospective study with various clinical cases is desirable, however it is uncommon to treat skin lesions within or near tattooed areas, but as tattoos increase in popularity radiation therapy treatment cases may increase as well.

## Conclusion

This study was motivated by an unexpected skin erythema in patient treated with an electron beam in the tattoo region. Although our findings represented in the dose perturbation parameter did not provide conclusive support to correlate them with the observed patient’s reaction, we believe it is still worth investigating the potential dosimetric effects of tattoo ink on patient’s treatment. For instance, as we mentioned in the paper, it is almost impossible to know for sure the amount of high-Z material present in the patient’s tattoo and its implications on the physiological changes in the skin of a tattooed area, which cannot be reproduced in an experiment without a biopsy. This study provides awareness that tattoo ink might produce dosimetric perturbations and therefore it should not be disregarded.
